# Effects of Different Ways of Music Stimulation on Exploring, Playing and Aggressive Behavior

**DOI:** 10.3390/ani15182721

**Published:** 2025-09-17

**Authors:** Mengyao Wu, Zhonghui Wang, Sitong Zhou, Xiaolong Zhang, Yunlong Zhao, Xuanning Liu, Bin Bai, Runze Liu, Honggui Liu, Wenzhong Zhao

**Affiliations:** 1 College of Animal Science and Technology, Northeast Agricultural University, No. 600 Changjiang Road, Harbin 150030, China; s230501016@neau.edu.cn (M.W.);; 2 Key Laboratory of Swine Facilities, Ministry of Agriculture and Rural Affairs, Northeast Agricultural University, No. 600 Changjiang Road, Harbin 150030, China; 3 Institute of New Rural Development, Northeast Agricultural University, No. 600 Changjiang Road, Harbin 150030, China

**Keywords:** animal welfare, weaned piglet, intermittent music, behavior, hippocampus, nervous substances

## Abstract

In the breeding of farm animals, music serves as an environmental enrichment factor that can improve the mood and welfare level of animals. However, it is not clear whether pigs receiving different modes of musical stimulation can improve cognitive performance. This study aimed to explore the extent to which different music stimulation methods affect the cognitive ability-related behaviors and neural substances of weaned piglets by providing them with various music stimuli. Fifty-four piglets were randomly divided into three groups: control group (C Group), continuous music group (CM group), and intermittent music group (IM group). The CM group received half an hour of music stimulation in the morning and afternoon of each day when the piglets were active, the IM group was given a cross-stimulation mode between the music playing time and the music pause time, and the C group had a music player installed in the enclosure, but no music was played, and the test period was 3 d. The results of the study showed the following: (1) Compared with piglets in the C group, piglets in the CM and IM groups showed more exploring behavior and less aggressive behavior (*p* < 0.05), while the playing behavior of piglets in the CM and IM groups was significantly higher than those in the C group (*p* < 0.05). (2) Compared with the CM group, the expression of cognition-related DCX, BDNF, and EGR1 genes in hippocampal tissues of the IM group was significantly higher (*p* < 0.05), and the expression of CREB was significantly lower (*p* < 0.05). (3) Western blot results showed that the protein expression of neural tissue development and cognitive-related genes (DCX and BDNF) in the hippocampal tissues of the IM group was significantly higher (*p* < 0.05), and the protein expression of EGR1 was highly significant (*p* < 0.01), compared with the CM group. These findings may indicate that intermittent music patterns can improve the cognitive abilities of weaned piglets regarding the surrounding physical and social environmental cognitive abilities.

## Simple Summary

In the breeding of farm animals, music, as a non-environmental enrichment factor, has shown potential in improving the health and welfare of animals. Piglets stimulated by continuous music and intermittent music showed more exploring behavior and less aggressive behavior. The expression levels of genes and proteins related to cognition in the hippocampus of piglets given intermittent music stimulation were higher than those of piglets given continuous music stimulation. This indicates that the intermittent music mode may improve the cognitive ability of weaned piglets to the surrounding physical and social environment to a certain extent. Its effect is better than continuous music.

## 1. Introduction

With the growing research on music, it has shown potential as a non-pharmacological intervention to improve animal health and welfare [1]. For example, in the music environment, the stereotyped behavior of sows raised in the crates and in the group is reduced [2]. Additionally, Papadakakis et al. (2019) also disclosed that music enrichment mitigated anxiety and depressive behaviors that appeared in adult rats [3], indicating that music can impact the behaviors of domesticated animals. Research indicates that cleverly interspersing 2 min music-free intervals in fast and slow tempo interwoven pieces has not only shown excellent results in treating cardiovascular disease in humans, but has also been more effective in lowering blood pressure, heart rate, and respiratory rate [4]. In animals, Kayleigh et al. (2019) showed that the provision of acoustic stimulation decreased the frequency of irregular behaviors (such as tongue rolling or vocalization) and even facilitated social interactions among cattle; however, it was also important to stop acoustic stimulation, which induced a deeper rest and enhanced ruminant behavior, which to some extent meant that the cattle achieved a deeper relaxation and were induced to show a higher degree of productivity [5], which suggests that alternating between music and no music may be a better approach. However, at this stage, there are fewer reports on the effects of intermittent music stimulation on behaviors such as exploration and social interaction of pigs, given that the cognitive ability of weaned piglets may affect their ability to adapt to new environments and peers. Whether different musical stimulation methods can enhance cognitive-related indicators, such as exploring, playing and social interaction behaviors of piglets, and reduce the weaning stress is a key scientific and technological issue in pig production.

As an enriched environment, music is regarded as a rich and appropriate factor in the study of neural plasticity related to cognitive ability [6,7]. Previous studies have shown that rodents exposed to music have a tendency to enhance neuroplasticity [8]. Kim et al. (2006) showed that hippocampal neurodevelopment was enhanced in rats exposed to music [9], and prenatal music-enriched environments promoted neural development in the motor cortex of young rats [10]. The above studies suggest that music has numerous benefits in the development of the brain nervous system [11], but it remains unclear whether different modes of musical stimulation affect the expression of cognitively related nerve factors and proteins in animals.

Brain-derived neurotrophic factor (BDNF) can promote the growth and differentiation of neurons and play an important role in the plasticity of neural structure and function [12]. The increased expression of BDNF plays an important role in the development of nerve cells [13]. The expression of BDNF in the hippocampus is also of great significance to improve animal memory and cognition [14]. This study endeavored to detect the expression of BDNF in the hippocampal tissues of weaned piglets under different modes of musical stimulation, and preliminarily explored the extent of its effect on the cognitive function of piglets. The expression of early growth responsive gene-1 (EGR1) in the brain is closely related to neuronal activity, and it is a key factor involved in the processes of neuronal salient plasticity, long-term memory formation, consolidation, and emotional response to stress and reward [15]. The doublecortin (DCX) gene encoding doublecortin, a microtubule-associated protein required for neuronal dissociation and cortical separation during cortical development, has been shown to be closely related to cognitive-related neuronal development in living organisms in past studies [16]. Therefore, the present study screened for both of the above metrics with the aim of preliminarily determining the effects of different modes of musical stimulation on factors related to cognitive function in weaned piglets.

In addition, cAMP response element-binding protein (CREB) is a key downstream regulator of the extracellular-response kinase signaling pathway, and CREB can directly regulate the expression of BDNF after it is activated [17,18]. However, it is not clear whether different music stimulation modes regulate CREB factors and cognitive neurodevelopment through BDNF and its downstream pathway. Therefore, this study used weaned piglets as the research object to explore the effects of different music stimulation methods on the mRNA level and protein expression of BDNF, DCX, EGR1, and CREB genes related to cognition in the hippocampus. This study investigates the extent of the effects of different music stimulation modes on cognition-related neurological substances in weaned piglets, and provides a reference for screening the optimal music stimulation modes for early life awareness-raising ability.

## 2. Materials and Methods

### 2.1. Animals and Management

**Figure 1:**
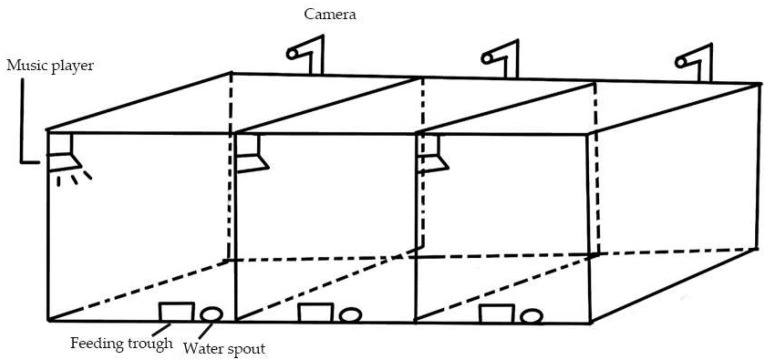
Stereogram of pig pig-rearing room. Sound insulation materials were attached around the room to improve the sound insulation effect.

This experimental site is the Northeast Agricultural University-Yabuli Pig Welfare Breeding Demonstration Base. Healthy 4-week-old weaned piglets of the Large White breed were chosen, and each rearing room was equipped with a pen against the wall, which was 3 m × 1.5 m × 1.2 m, with a slatted floor, and the house was fitted with a feeding trough and a water spout, as shown in Figure 1. Based on prior laboratory research, a more sensitive combination of musical tempo and pitch was selected: Mozart’s Sonata for Two Pianos in D major K.448 with a fast tempo (200 bpm) and a two-octave increase in pitch (music intensity of 60–70 dB). All piglets had their teeth clipped and castrated within three days after birth. Transcribe v8.71 software (Seventh String Software, London, UK) was used to adjust the rhythm and tone of the original Mozart music. The music player position and music intensity were kept constant throughout the test. Following a one-week acclimation period, different groups of piglets received distinct musical stimuli from 9:00 a.m. to 9:30 a.m. and 2:30 p.m. to 3:00 p.m. over a total of 3 days. During the whole experiment, two breeders managed piglets together and strictly followed the production management requirements. The health status of piglets was routinely checked and recorded every day. The pig house was cleaned and disinfected regularly. The automatic ventilation device was installed in the house. The temperature of the pig house was controlled at (23 ± 2) °C, the relative humidity was controlled at 65–70%, and all piglets were free to feed and drink.

### 2.2. Experimental Animal Grouping and Sample Collection

The sampling procedures were followed in accordance with the Guidelines on Ethical Treatment of Experimental Animals (2006) (No. 398) by the Ministry of Science and Technology, China. At the end of the experimental period, the piglets were euthanized humanely after 12 h of fasting to collect tissue samples. First, piglets were deeply sedated by intramuscular injection of xylazine (2.0 mg/kg). After loss of consciousness was confirmed, a lethal dose of pentobarbital sodium (100 mg/kg) was injected through the ear vein. Death was confirmed by the inability to hear the heartbeat for more than 5 min, lack of corneal reflex, and lack of spontaneous breathing activity.

Fifty-four weaned piglets with an initial weight of (6.9 ± 0.45 kg) were randomly divided into 3 groups, each group had 3 pens, and each pen had 6 piglets (3 males, 3 females). Each piglet was ear-tagged for subsequent observation. The groups were designated as the control group (C group), the continuous music group (CM group), and the intermittent music group (IM group, where the music playback period was equal to the pause time). These piglets were reared in 3 identical and soundproof feeding rooms.

At the end of the experiment, 6 piglets (3 males, 3 females) were randomly selected from each group for euthanasia. The pig skulls were opened via a prismatic cross incision, and the whole brain tissue was rapidly removed and placed on a sterile ice box for dissection. The hippocampal tissues were rapidly frozen in liquid nitrogen and then stored in a refrigerator at −80 °C for subsequent real-time fluorescence quantitative PCR (qRT-PCR) and Western blot detection.

### 2.3. Behavior Video Capture

The behavior of piglets in each test group was recorded using a Hikvision HD video camera mounted on the upper left side of the test room, allowing for panoramic observation. Behavior observations were conducted during the daily music testing sessions (9:00 a.m. to 9:30 a.m. and 2:30 p.m. to 3:00 p.m.). Event behaviors (exploring, playing, and aggressive) were observed using the instantaneous sampling observation method at 20 s intervals [19] and recorded using the 1–0 sampling method to document the total number of these behaviors during observation. The behavioral parameters observed in this study are shown in Table 1 [20].

### 2.4. Real-Time Quantitative PCR (qRT-PCR)

Target genes were detected in the hippocampal tissues of piglets from the C, CM, and IM groups, with six biological replicates for each group. The sequences of BDNF, DCX, EGR1, TRKB, CREB, PDGF, and NGFR related to neural tissue development and cognition were designed and synthesized according to the published pig gene sequences in GenBank. The primer sequences are shown in Table 2. The extraction and reverse transcription of total RNA in the hippocampal tissues of piglets were performed strictly according to the instructions, using RNAiso Plus (Taraka, Kyoto, Japan) and Reverse transcription on Kit (Toyobo, Osaka, Japan). The qRT-PCR reaction system consisted of 10 μL containing 5 μL of fluorescent dye, 1 μL of cDNA substrate, 0.3 μL of each forward and reverse primer, and 3.4 μL of double-distilled water. Reaction conditions were as follows: pre-denaturation at 95 °C for 1 min, 95 °C for 20 s, 60 °C for 5 s, and a total of 40 cycles. The relative expression of each gene was calculated according to the 2^−∆∆CT^ method [21], with β-actin serving as the internal reference gene. The full name and main function of the gene are shown in Table S1.

### 2.5. Western Blot

This detection method was based on the experimental procedure of Han et al. [22]. The mixture of protein lysate (Beyotime, Shanghai, China) and PMSF (Beyotime, Shanghai, China) was added to the hippocampus for grinding at low temperature. The protein concentration was determined by the BCA protein concentration detection kit (Biosharp, Beijing, China) and then diluted to the same concentration. The target protein was obtained by SDS-PAGE and transferred to a PVDF membrane. The membrane was blocked in skimmed milk for 2 h and then incubated with the primary antibody for 18 h. The membrane was rinsed and then incubated in the secondary antibody for 1 h. The signal was displayed using an ultrasensitive ECL solution (Biosharp, Beijing, China). The optical density (OD) value of the protein bands was displayed using the Image J 1.53t software (National Institutes of Health, Bethesda, MD, USA). The antibodies used for the Western blot are shown in Table 3.

### 2.6. Statistical Analysis

All results were statistically analyzed using SPSS 27.0. Behavioral data were analyzed using two-way ANOVA with the statistical model:

*Y*_ij_ = *µ* + *A*_i_ + *B*_j_ + (*AB*)_ij_ + e

where “*Y*_ij_” is the target trait, *µ* is the overall mean, “*A*_i_” is the effect of different stimulus modalities (3 levels), “*B*_j_” is the effect of days of testing (3 levels), “(*AB*)_ij_” is the interaction, and “e” is the random error. One-way ANOVA was used for PCR and Western blot data. All results are expressed as mean ± standard error (SE). *p* value < 0.05 indicated a significant difference between groups, *p* value < 0.01 indicated a very significant difference, and *p* value < 0.001 indicated an extremely significant difference.

## 3. Results

### 3.1. Effects of Different Music Stimulation Methods on the Exploring Behavior of Piglets

As shown in Table 4, the interaction between different test groups and testing days had a significant effect on the exploring behavior in piglets (*p* < 0.05). Testing days significantly influenced the exploring behavior of piglets, with the first day showing significantly higher exploring behavior than Day 3 (*p* < 0.05). There was no significant difference in exploring behavior between Day 1 and Day 2 or between Day 2 and Day 3 (*p* > 0.05). Different experimental groups had significant effects on the exploring behavior of piglets (*p* < 0.05). Specifically, the exploring behavior of piglets in the IM group, CM group, and C group decreased significantly in that order (*p* < 0.05).

On any test day, exploring behavior in piglets decreased significantly in the order of IM group, CM group, and C group (*p* < 0.05). In the IM group, there was no significant difference between Day 1 and Day 2, but both were significantly higher than Day 3 (*p* < 0.05). The CM group showed a significant decrease over the test days (*p* < 0.05), while the C group remained unchanged (*p* > 0.05).

### 3.2. Effects of Different Music Stimulation Methods on the Playing Behavior of Piglets

The playing behavior of piglets is shown in Table 5. The interaction between test groups and test days had no significant effect on playing behavior (*p* > 0.05). The number of test days significantly affected the playing behavior (*p* < 0.05). The playing behavior of piglets was significantly higher on Day 1 compared to Day 3 (*p* < 0.05), with no significant differences between other days (*p* > 0.05). Experimental groups had a significant effect on the expression of piglet playing behavior (*p* < 0.05). Compared with the C group, the IM and CM groups were significantly increased (*p* < 0.05), and there was no significant difference between the IM and CM groups (*p* > 0.05).

On any test day, there was no significant difference in the expression of playing behavior between the IM group and the CM group (*p* > 0.05), but they were significantly higher than that of the C group (*p* < 0.05). The expression of playing behavior of piglets in the IM and CM groups on Day 1 was significantly higher than that on Day 3 (*p* < 0.05), with no significant differences between other days. There was no significant difference in the C group during the test (*p* > 0.05).

### 3.3. Effects of Different Music Stimulation Methods on the Aggressive Behavior of Piglets

The aggressive behavior of piglets is shown in Table 6. The interaction between different test groups and test days significantly affected the aggressive behavior of piglets *(p* < 0.05). Test days also significantly influenced aggressive behavior (*p* < 0.05), with Day 1 being significantly higher than Day 2 and Day 3 (*p* < 0.05), but there was no significant difference between Day 2 and Day 3 (*p* > 0.05). Experimental groups significantly affected aggressive behavior (*p* < 0.05), with the C, CM, and IM groups showing a significant decrease in that order (*p* < 0.05).

On any test day, the expression of aggressive behavior of piglets in the C, CM, and IM groups decreased significantly in turn (*p* < 0.05). There was no significant difference between Day 1 and Day 3 for the CM group (*p* > 0.05), but both were significantly higher than Day 2 (*p* < 0.05). There was no significant difference between the C and IM groups during the whole test period (*p* > 0.05).

### 3.4. mRNA Level of Neurodevelopment and Cognition in the Hippocampus

**Figure 2:**
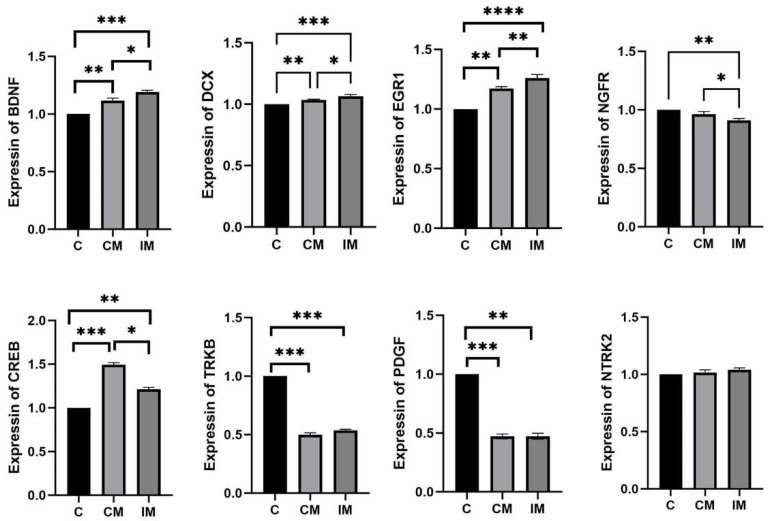
The mRNA level of neurotrophic and cognitive-related genes in the hypothalamus. Results are presented as mean ± SE. * *p* < 0.05, ** *p* < 0.01, *** *p* < 0.001, **** *p* < 0.0001. Absence of * indicates no significance.

The mRNA level of neurodevelopment and cognition in the hippocampus tissues is shown in Figure 2. Compared with the C group, the mRNA level of BDNF significantly increased in the CM and IM groups (*p* < 0.01), with the IM group showing significantly higher expression than the CM group (*p* < 0.05). Compared with the C group, the mRNA level of DCX significantly increased in the CM and IM groups (*p* < 0.01), with the IM group exhibiting significantly higher expression than the CM group (*p* < 0.05). The mRNA level of EGR1 significantly increased in turn in the C, CM, and IM groups (*p* < 0.05). The mRNA level of NGFR was significantly decreased in turn in the C, CM, and IM groups (*p* < 0.05). Compared with the C group, the mRNA level of CREB in the CM and IM groups increased significantly (*p* < 0.05), and the mRNA level of the CM group was significantly higher than that of the IM group (*p* < 0.05). The mRNA level of TRKB was significantly decreased in the CM and IM groups compared to the C group (*p* < 0.001), with no significant difference between the CM and IM groups (*p* > 0.05). Compared with the C group, the mRNA level of PDGF was significantly decreased in the CM and IM groups (*p* < 0.01 for CM, *p* < 0.001 for IM), with no significant difference between the CM and IM groups (*p* > 0.05). There was no significant difference in the mRNA level of NTRK2 among the C, CM, and IM groups (*p* > 0.05).

### 3.5. Protein Expression Related to Neurodevelopment and Cognition in the Hippocampus

**Figure 3:**
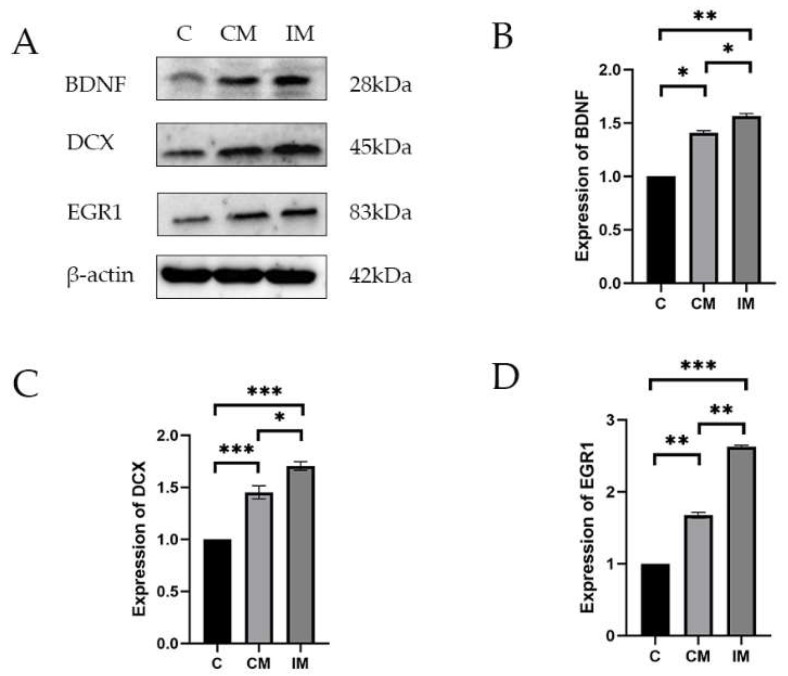
The protein expression of emotion-related genes in the hippocampus. (**A**) The protein expression of BDNF, DCX, and EGR1 in hippocampal tissues. (**B**–**D**) The protein density was quantified using Image J software. BA means baicalin (n = 3). Results are presented as mean ± SE. * *p* < 0.05, ** *p* < 0.01, *** *p* < 0.001. Absence of * indicates no significance.

The protein expressions of POMC, NR3C1, and FKBP5 in the hippocampus tissues are shown in Figure 3. The expressions of BDNF, DCX, and EGR1 in the hippocampus of piglets in the IM, CM, and C groups were consistent with the results of mRNA. Compared with the C group, the expression of BDNF was significantly increased in the CM group (*p* < 0.05) and even more so in the IM group (*p* < 0.001), with the IM group showing significantly higher expression than the CM group (*p* < 0.05). Compared with the C group, the expression of DCX was significantly increased in the CM and IM groups (*p* < 0.001), with the IM group exhibiting significantly higher expression than the CM group. Compared with the C group, the expression of EGR1 was significantly increased in the CM group (*p* < 0.01) and even more so in the IM group (*p* < 0.001), with the IM group showing significantly higher expression than the CM group (*p* < 0.01).

## 4. Discussions

Based on the expression of animal behavior, we can gain insights into not only the physical condition of animals but also into their cognitive development and abilities. This study revealed that varying conditions of musical stimulation altered piglet behavior, such as exploring and playing behavior. The exploring behavior was closely linked to cognitive function, and prior research has noted that cognitive enhancement and emotional stability may significantly reduce the activity time of pigs in unfamiliar environments, while their exploring behavior increased significantly when there were known and familiar companions, which was similar to the performance of pigs taking decompression drugs [23]. In this experiment, compared to the C group, both the IM and CM groups exhibited significantly increased exploring behavior, with the IM group surpassing the CM group. However, this effect diminished over prolonged musical stimulation. This study hypothesized that intermittent musical stimulation enhances memory and emotional stability in weaned piglets, but with a time-limited effect. Furthermore, intermittent stimulation, as a novel environmental enrichment, may better satisfy the psychological needs of piglets (e.g., curiosity-driven environmental exploration), thereby yielding more positive behavioral outcomes.

The playing behavior in the CM and IM groups was significantly higher than that in the C group, suggesting that music could enhance social contact among piglets, which may be an ability that can only be demonstrated after the cognitive development of piglets has reached a certain level. Combined with the results of the expression of cognition-related genes and proteins in the hippocampal tissues, this study hypothesized that music may be useful for a short period of time to promote cognition-related behavioral performance and neurodevelopment in weaned piglets. However, during the whole test period, there was no significant difference in the playing behavior of piglets between the IM and CM groups, which was not consistent with our overall research trend. It may be related to the fact that the IM group devotes more energy to exploring the environment, which can be confirmed by the fact that the exploring behavior of the IM group was much higher than other groups. Unfortunately, this study did not count or compare the occurrence of the behavior of the weaned piglets of the IM group between exploring their physical surroundings and playing with peers. Future experiments will be designed to determine this subtle difference.

While the exploring behavior in the IM and CM groups declined over time, it remained significantly higher than that in the C group. This contradicts findings from prior studies, where prolonged musical stimulation reduced playfulness in music-exposed groups, with no significant environmental variation [24]. This study hypothesized that the reason for the difference may arise from our use of short-term musical stimulation, and the positive effect of music had not yet decreased significantly along with the extension of time. Additionally, the number of exploring behaviors exhibited by piglets in the IM group was significantly higher than that of piglets in the CM group, suggesting that the effect of intermittent stimulation was still affecting the piglets, but the duration of this effect remains unclear due to experimental constraints.

The relatively low incidence of piglet fighting in this study may primarily be attributed to the one-week acclimation period prior to trial initiation. Aggressive behavior is an important means for pigs to determine their social order. During the previous week of rearing, the social sequence of the piglets was relatively fixed. Throughout the testing period, both the CM and IM groups exhibited significantly fewer aggressive behaviors compared to the C group. This aligns with the results of a previous study [25], suggesting that both intermittent and continuous music may promote the cognitive ability of piglets, facilitate the rapid formation of social sequences, and reduce the number of aggressive behaviors. Notably, aggressive behaviors in the IM group demonstrated a progressive decline over time, whereas the CM group showed an initial reduction followed by a resurgence. This may be related to the fact that continuous music stimulation accelerated the adaptation process of weaned piglets. Subsequently, with increase in age-related breeding density and resource competition led to a slight increase in the aggressive behaviors of piglets in the CM group.

In this experiment, cognition and neurodevelopment-related genes within the hippocampal tissue as potential research targets were selected as research targets based on observed behavioral changes in piglets. In the past, hippocampal tissue has often been used as a means to establish cognitive and emotion-related models [26]. Our findings demonstrated that both the IM and CM groups exhibited significantly higher BDNF gene and protein expression levels in hippocampal tissues compared to the C group, with the IM group showing markedly greater upregulation than the CM group. This aligns with existing evidence that environmental enrichment, including musical stimulation [27,28,29], toys [3], and complex habitats [30], enhances the expression of BDNF across brain regions, correlating with reduced aberrant behaviors. In combination with the results of the exploring and playing behaviors, this suggests that the intermittent music group can improve the positive behaviors of piglets. The elevated BDNF levels in the IM group, combined with their superior exploring and playing behaviors, suggest that intermittent auditory enrichment may optimize positive behavioral outcomes. From a neurodevelopmental perspective, BDNF plays a critical role in early cognitive development, further supporting the hypothesis that its upregulation under intermittent stimulation reflects enhanced cognitive capacities in piglets.

Notably, the expression of EGR1 displayed a graded increase across groups (C < CM < IM), consistent with its established involvement in neuronal plasticity, memory consolidation, and stress response modulation [15]. It has been noted that the expression of EGR1 was significantly decreased in the hippocampal tissues of mice exposed to 14 days of chronic stress, accompanied by cognitive deficits [31]. In addition, studies have shown that after social isolation stimulation, the expression of EGR1 in the hippocampus and hypothalamus of male mice also decreased significantly and showed more anxiety behavior [32]. In music-stimulated piglets, the continued increase in the expression of EGR1 means improved cognitive flexibility in managing environmental changes, but mechanism validation requires further study.

The DCX gene encodes doublecortin, a microtubule-associated protein essential for neuronal migration and cortical laminar organization during cerebral cortical development [16]. Our results showed that the expression of DCX in the music group was significantly higher than that in the C group, and among different music groups, the IM group was also significantly higher than the CM group, which was consistent with the previous research results. In the past studies, it was generally believed that positive stimulation could increase the expression of DCX in the hippocampus, and the expression of DCX in mice living in an enriched environment was significantly increased [33]. Some studies have also pointed out that the expression of DCX in laying hens living outdoors for a long time is much lower than those living indoors. The researchers believe that the reason for this phenomenon may be that laying hens living outdoors have greater survival pressure and challenges [34]. This indicates that intermittent music has a better stimulating effect on piglets than continuous music, allowing them to be in a better welfare and cognitive state, thereby exhibiting more positive behaviors and stronger adaptability to the environment.

## 5. Conclusions

In summary, intermittent music stimulation could promote the development of cognitive and nervous system functions and behavioral responses in piglets. However, at this stage, there are relatively few studies on the cognitive effects of intermittent music stimulation on weaned piglets. We cannot systematically discuss the changes in neurophysiological responses of piglets to intermittent music stimulation, and how these changes will affect other cognitive indicators of piglets. This requires further research in the future to clarify its mechanism.

## Figures and Tables

**Table 1:** The behavioral categories and definitions of piglets.

Behavioral Categories	Behavior Definition
Event behavior	
Exploring	Nose sniffing, touching, or arching on the floor; nose or mouth sniffing, arching, gnawing, or biting ring bar and chute.
Playing	Turning and jumping, suddenly lying down; the target pig is close to the other pig, sniffing, arching the neck and back, with the nose; pigs place two front legs on the front or back of a companion.
Aggressive	Two piggy heads pushing, fighting, biting, banging, or pushing each other head-to-head.

**Table 2:** Gene-specific primers for qRT-PCR.

Gene	Serial Number	Primer Sequence (5′ to 3′)
BDNF	NM_214259.2	Forward: GAACTCCCAGTGCCGAACTACC
		Reverse: CCTTATGAACCGCCAGCCAATTC
DCX	XM_013986335.2	Forward: ATGCTCTCCTGGCTGACCTGAC
		Reverse: AGCTCATCCATGCTTCCAATCTTCC
EGR1	XM_003123974.6	Forward: AGTTTGCCAGGAGCGATGAA
		Reverse: AGGCCACACTTTTGTCTGCT
TRKB	XM_021064645.1	Forward: GACGCTGAAGGATGCCAGTGAC
		Reverse: AGACGCCATAGAACTTGACGATGTG
CREB	WP_185668273.1	Forward: CACCTGCCATCACCACTGTAACG
		Reverse: CTGAATTGCTCCTCCCTGGGTAATG
PDGFA	XM_021085925.1	Forward: ACGGGCTCCAGCAGTTCTACC
		Reverse: CCACCAGGTCCGAGGAGTCTATG
NGFR	XM_021067136.1	Forward: GGAGGTGGAGAAGCTGCTGAATG
		Reverse: AGTCTATGTGCTCGGGCTGGTAG
β-actin	NM_001170517.2	Forward: GGCACCACACCTTCTACAACGAG
		Reverse: TCATCTTCTCACGGTTGGCTTTGG

**Table 3:** Antibodies and dilutions used for Western blot.

Antibody	Dilution Multiple	kDa	Source	Producer
BDNF	1:1000	28	rabbit	WANLEIBIO, Shenyang, China
DCX	1:1000	40–45	rabbit	WANLEIBIO, Shenyang, China
EGR1	1:1000	82	rabbit	WANLEIBIO, Shenyang, China
β-actin	1:5000	42	rabbit	Beyotime, Shanghai, China

**Table 4:** Effects of combined music under different stimulation methods on the exploring behavior of piglets.

Group	Day 1(n)	Day 2(n)	Day 3(n)	Main Effect (Grouping)
C group	56.67 ± 1.43 ^c^	55.33 ± 1.20 ^c^	54.33 ± 0.71 ^c^	55.44 ± 0.67 ^c^
CM group	77.16 ± 1.92 ^bx^	70.50 ± 1.09 ^by^	59.33 ± 0.84 ^bz^	69.00 ± 1.93 ^b^
IM group	86.17 ± 2.39 ^ax^	82.50 ± 1.43 ^ax^	75.50 ± 1.18 ^ay^	81.38 ± 1.43 ^a^
Main effect (test days)	73.33 ± 3.18 ^x^	69.44 ± 2.78 ^xy^	63.06 ± 2.25 ^y^	--
--	Test days	Grouping	Test days × Grouping	--
Group	Day 1	Day 2	Day 3	Main effect (grouping)
P ratio	<0.001	<0.001	<0.001	--
F ratio	38.847	242.916	7.489	--

Note: ^a^, ^b^, and ^c^ denote significant differences between different stimulation methods on the same test day (*p* < 0.05); ^x^, ^y^, and ^z^ denote significant differences between the same stimulation method on the same test day (*p* < 0.05); the same letter or no letter indicates no significant difference (*p* > 0.05); and ^x^, ^y^, and ^z^ denote significant differences between the same test day (*p* < 0.05). Results were presented as means ± SE, n = 6.

**Table 5:** Effects of combined music under different stimulation methods on the playing behavior of piglets.

Group	Day 1(n)	Day 2(n)	Day 3(n)	Main Effect (Grouping)
C group	20.00 ± 1.15 ^b^	19.17 ± 0.40 ^b^	20.00 ± 1.03 ^b^	19.11 ± 0.46 ^b^
CM group	30.33 ± 1.45 ^ax^	27.17 ± 0.83 ^axy^	25.17 ± 0.75 ^ay^	27.56 ± 0.77 ^a^
IM group	32.33 ± 1.33 ^ax^	28.33 ± 1.12 ^ay^	27 ± 0.93 ^ay^	29.22 ± 0.83 ^a^
Main effect (test days)	27.56 ± 1.49 ^x^	24.89 ± 1.09 ^xy^	23.44 ± 1.01 ^y^	--
--	Test days	Grouping	Test days × Grouping	--
P ratio	<0.001	<0.001	0.352	--
F ratio	12.985	87.727	1.136	--

Note: ^a^ and ^b^ show significant differences between different stimulation methods on the same test day (*p* < 0.05); ^x^ and ^y^, show significant differences between the same stimulation method on the same test day (*p* < 0.05); the same letter or no letter indicates no significant difference (*p* > 0.05). The results are shown as mean ± SE. Group C, the control group; CM group, continuous music group; IM group, interval music group, n = 6.

**Table 6:** Effects of combined music under different stimulation methods on the aggressive behavior of piglets.

Group	Day 1(n)	Day 2(n)	Day 3(n)	Main Effect (Grouping)
C group	11.33 ± 0.71 ^a^	10.83 ± 0.80 ^a^	11.00 ± 1.21 ^a^	9.56 ± 0.58 ^a^
CM group	8 ± 0.37 ^bx^	7 ± 0.45 ^by^	7.83 ± 1.07 ^bx^	6.44 ± 0.35 ^b^
IM group	5.67 ± 0.33 ^c^	5.17 ± 0.48 ^c^	4.33 ± 0.61 ^c^	5.06 ± 0.30 ^c^
Main effect (grouping)	8.72 ± 0.73 ^x^	6.61 ± 0.45 ^y^	5.72 ± 0.41 ^y^	
	Test days	Grouping	Test days × Grouping	
P ratio	<0.001	<0.001	0.001	
F ratio	33.449	74.797	5.254	

Note: ^a^, ^b^ and ^c^ denote significant differences between different stimulation methods on the same test day (*p* < 0.05); ^x^ and ^y^, denote significant differences between the same stimulation method on the same test day (*p* < 0.05); the same letter or no letter indicates no significant difference (*p* > 0.05); and ^x^ and ^y^ denote significant differences between the same test day (*p* < 0.05). Results were presented as means ± SE. Group C, control group; CM group, continuous music group; IM group, interval music group, n = 6.

## Data Availability

The raw data that support the conclusion of this article will be provided by the authors upon request.

## Supplementary Materials

The following supporting information can be downloaded at: https://www.mdpi.com/article/10.3390/ani15182721/s1, Table S1. Primers were used to detect genes related to neurotrophy and cognition in the hippocampus of piglets; protein bands.
