# Supplementary material for: Effects of Different Ways of Music Stimulation on Exploring, Playing and Aggressive Behavior
Source: Animals (Basel). 2025 Sep 17;15(18):2721. doi: 10.3390/ani15182721 (PMC12466348; doi:10.3390/ani15182721)
Supplement: Supplementary file 1 [file animals-15-02721-s001.zip › animals-3796056-Table S1.pdf]

Table S1. Primers were used to detect genes related to neurotrophy and cognition in the hippocampus of piglets.

Gene	Full name	Primary functions
BDNF	Brain-derived neurotrophic factor	A key protein that supports the survival, growth, and differentiation of neurons. It plays a central role in synaptic plasticity, which is essential for learning and memory.
DCX	Doublecortin	A microtubule-associated protein that is critically involved in neuronal migration and the regulation of neurogenesis (the birth of new neurons). It is a classic marker for immature neurons.
EGR1	Early growth response factor	An immediate-early gene and transcription factor that is rapidly activated in response to various stimuli. It is crucial for synaptic plasticity, memory formation, and neuronal development.
TRKB	Tyrosine kinase receptor b	The high-affinity receptor for BDNF. When activated by BDNF, it triggers intracellular signaling pathways that promote neuronal survival, growth, and synaptic strengthening.
CREB	Camp-response element binding protein	A fundamental transcription factor that regulates the expression of many genes (including BDNF and Egr1) involved in neuronal plasticity, survival, and long-term memory formation.

PDGFA	Platelet derived growth factor subunit A	While important in blood vessel formation (angiogenesis), it also plays a significant role in the central nervous system by promoting the proliferation and survival of oligodendrocyte progenitor cells, which are crucial for myelination.
NGFR	Nerve growth factor receptor	A receptor that binds to various neurotrophins (including BDNF and NGF). It can promote both neuronal survival and apoptosis (cell death), depending on the cellular context. It is also involved in regulating myelination.
β-actin	Beta-actin	Most commonly used as a housekeeping gene in molecular biology experiments (e.g., qPCR, Western Blot) for normalizing data due to its constitutive and stable expression across many cell types and conditions.
