# Supplementary figures and images for: Effects of Different Ways of Music Stimulation on Exploring, Playing and Aggressive Behavior
Source: Animals (Basel). 2025 Sep 17;15(18):2721. doi: 10.3390/ani15182721 (PMC12466348; doi:10.3390/ani15182721)

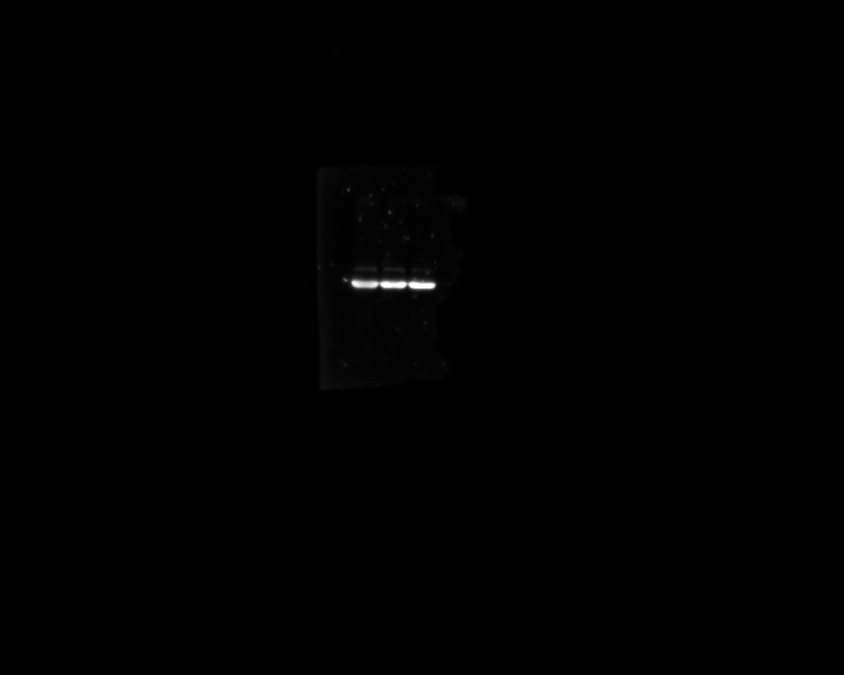

Supplement: Supplementary file 1 [file animals-15-02721-s001.zip › 2025-06-19-081600-image12-sub0-As-Displayed.tif]

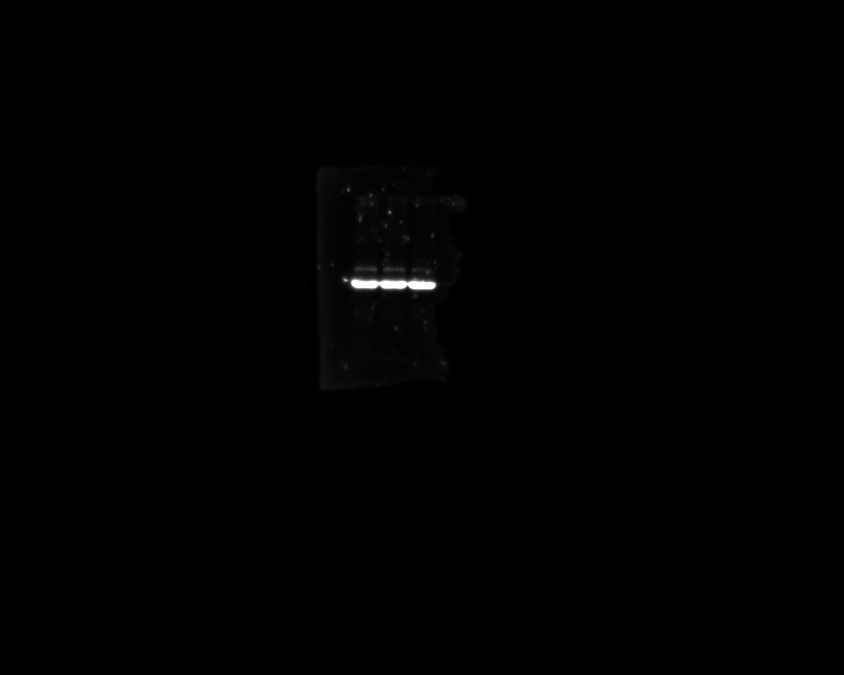

Supplement: Supplementary file 1 [file animals-15-02721-s001.zip › 2025-06-19-081600-image12-sub1-As-Displayed.tif]

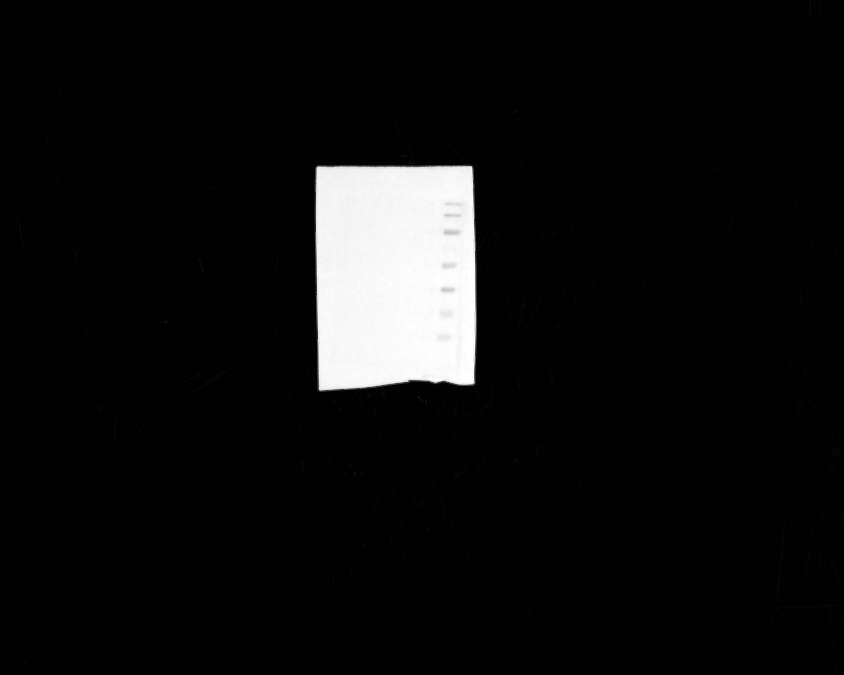

Supplement: Supplementary file 1 [file animals-15-02721-s001.zip › 2025-06-19-081600-image12-sub2-As-Displayed.tif]

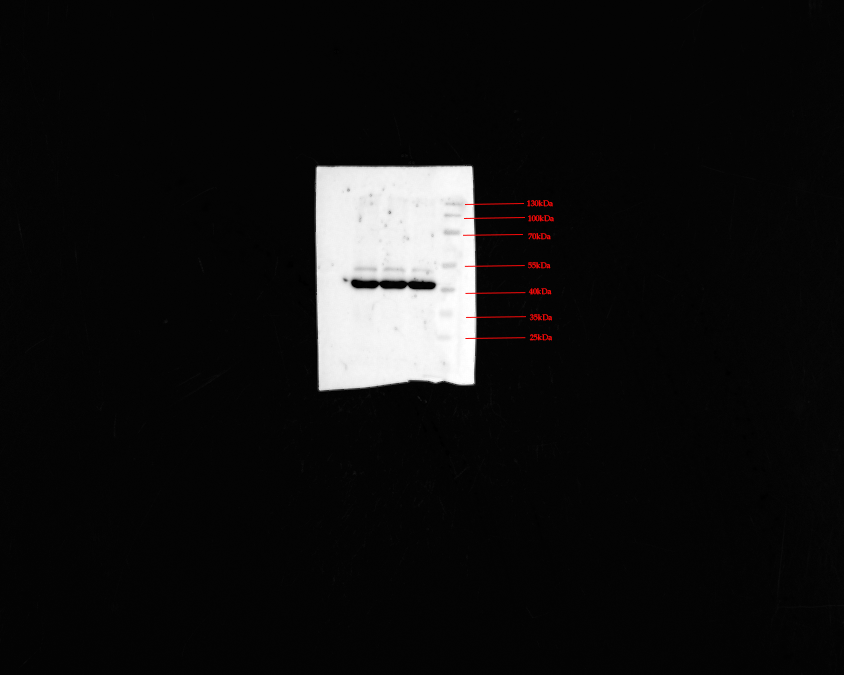

Supplement: Supplementary file 1 [file animals-15-02721-s001.zip › 2025-06-19-081633-image12-42kda.tif]

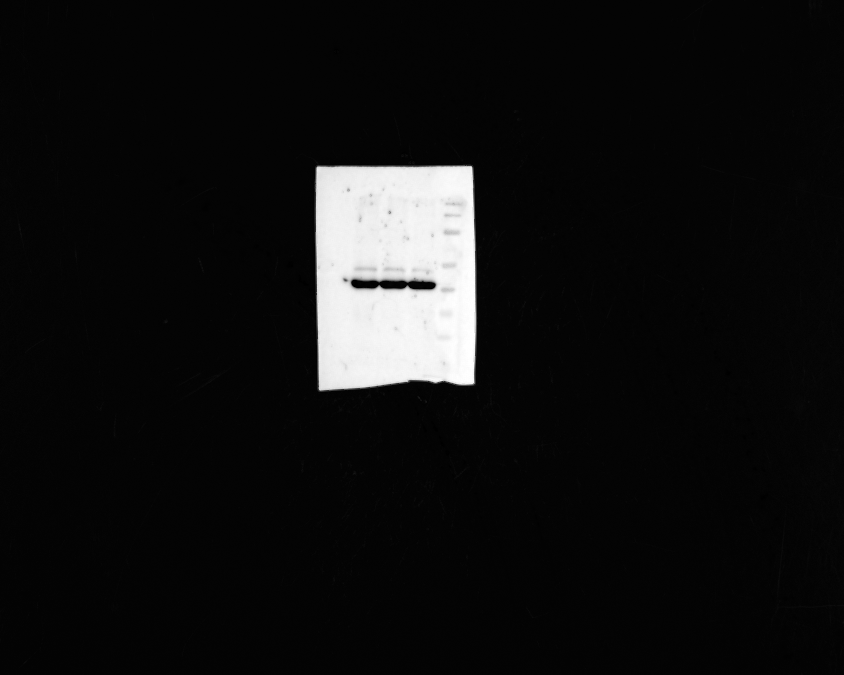

Supplement: Supplementary file 1 [file animals-15-02721-s001.zip › 2025-06-19-081633-image12.tif]

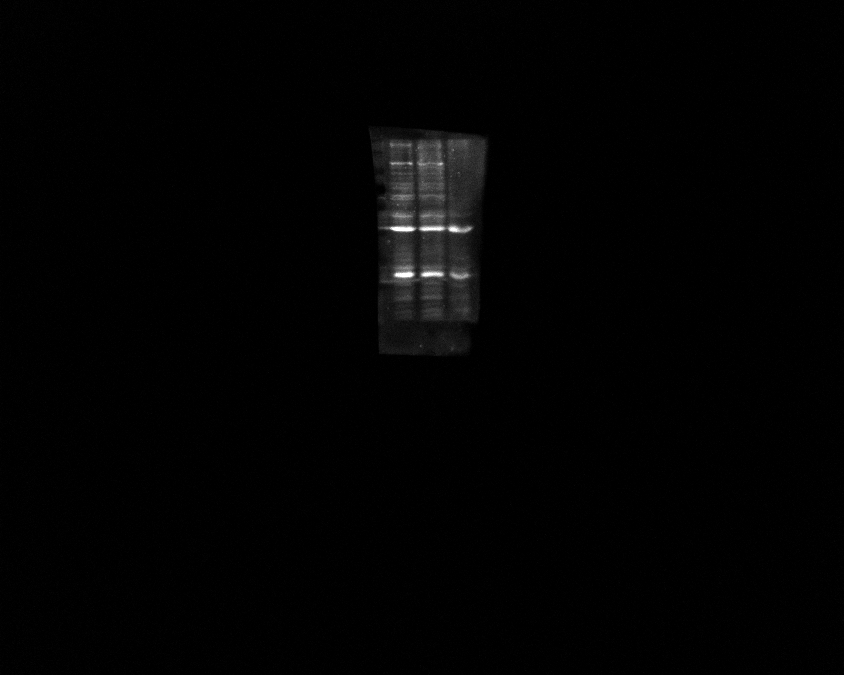

Supplement: Supplementary file 1 [file animals-15-02721-s001.zip › 2025-06-19-102642-image13-sub0-As-Displayed.tif]

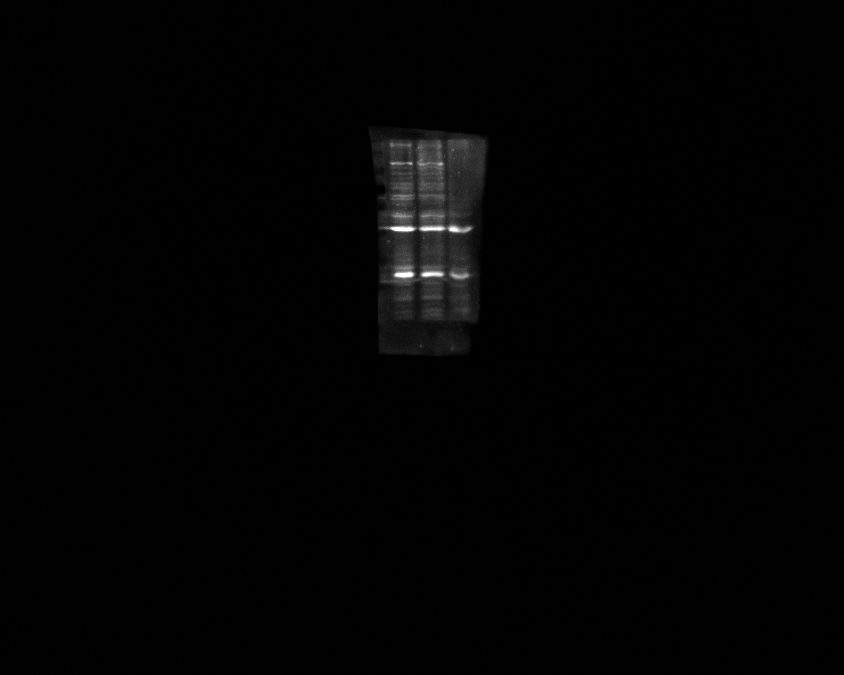

Supplement: Supplementary file 1 [file animals-15-02721-s001.zip › 2025-06-19-102642-image13-sub1-As-Displayed.tif]

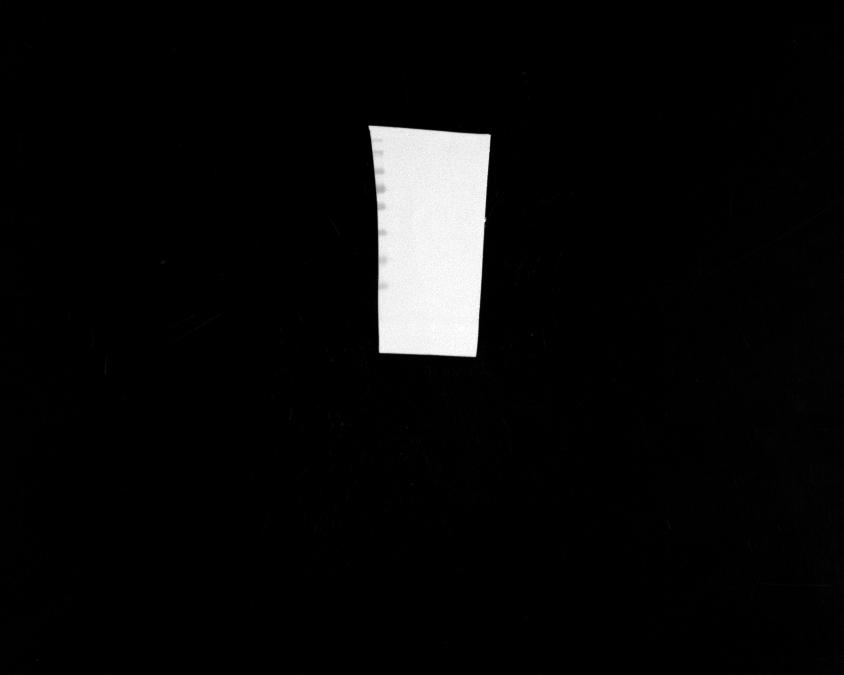

Supplement: Supplementary file 1 [file animals-15-02721-s001.zip › 2025-06-19-102642-image13-sub2-As-Displayed.tif]

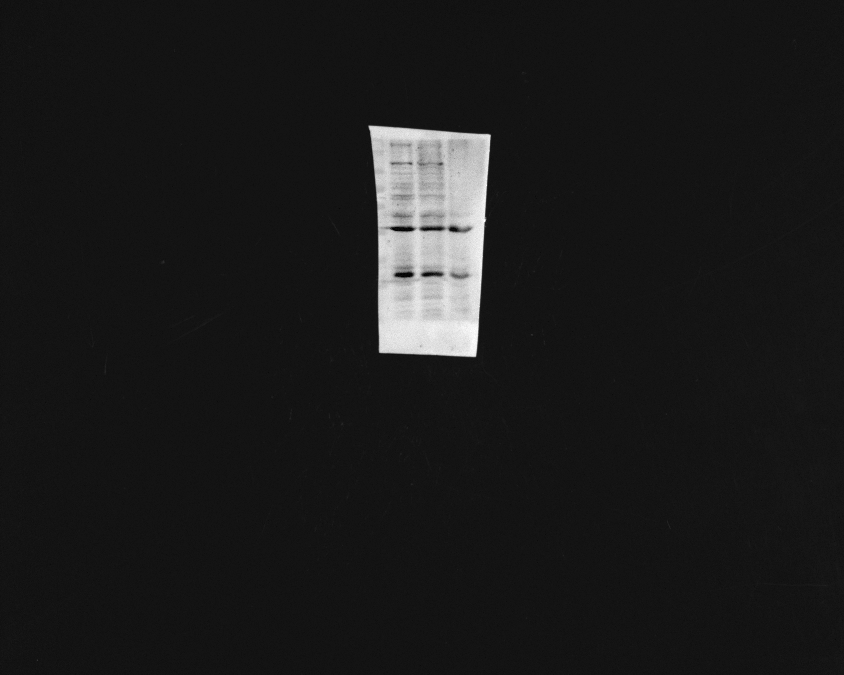

Supplement: Supplementary file 1 [file animals-15-02721-s001.zip › 2025-06-19-102655-image13 25-35kd.tif]

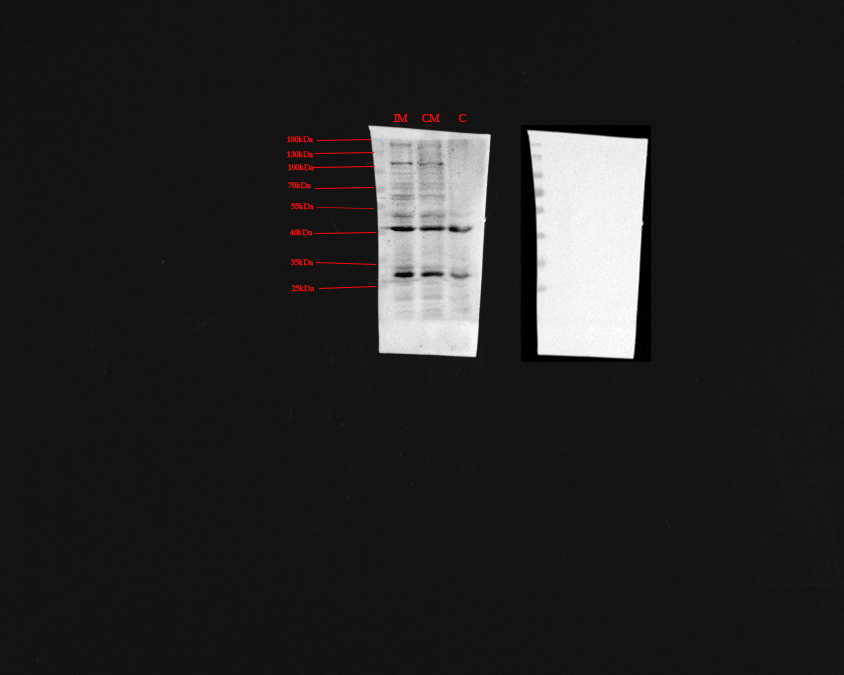

Supplement: Supplementary file 1 [file animals-15-02721-s001.zip › 2025-06-19-102655-image13 28kd.tif]

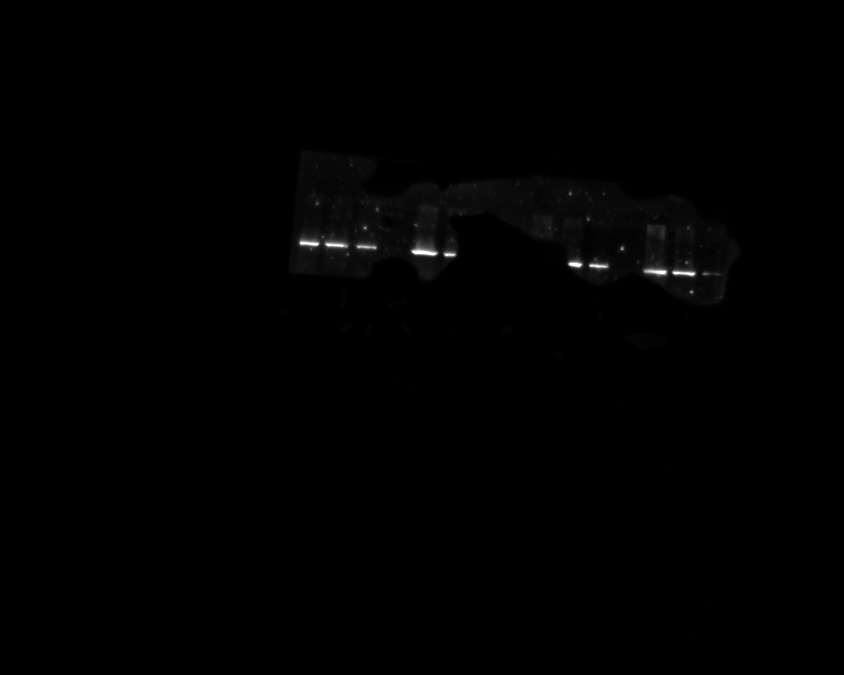

Supplement: Supplementary file 1 [file animals-15-02721-s001.zip › 2025-06-21-080738-image5-sub0-As-Displayed.tif]

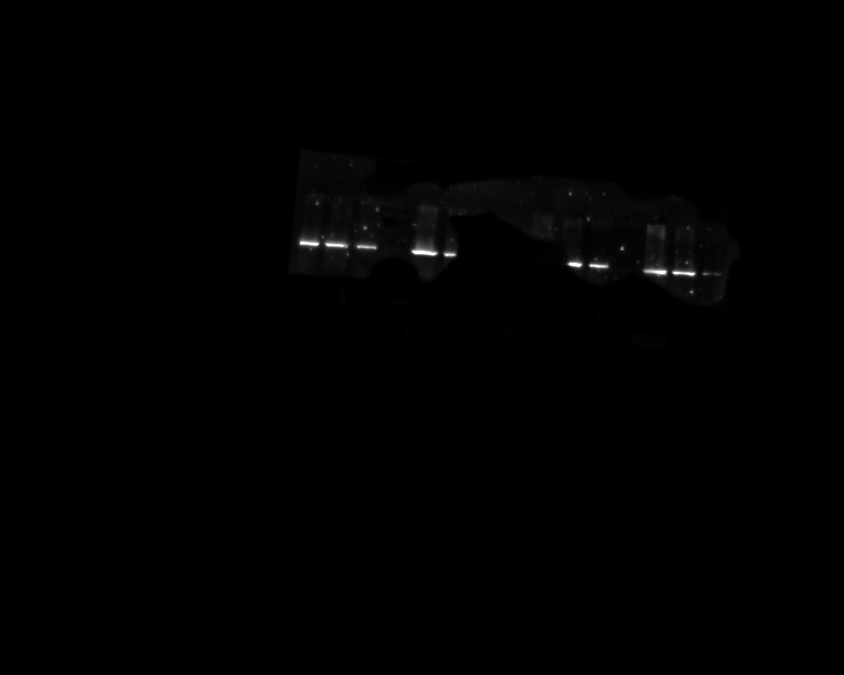

Supplement: Supplementary file 1 [file animals-15-02721-s001.zip › 2025-06-21-080738-image5-sub1-As-Displayed.tif]

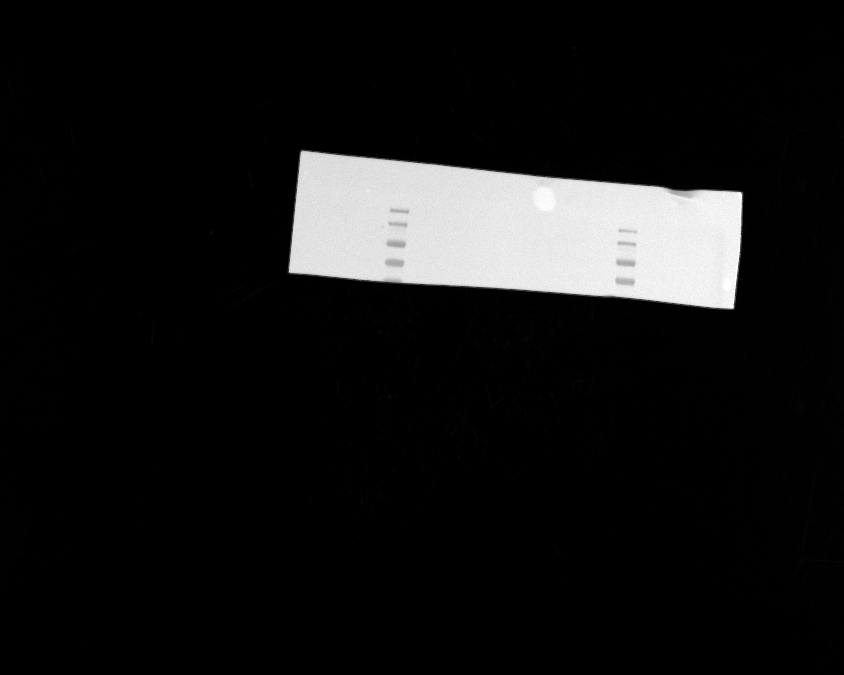

Supplement: Supplementary file 1 [file animals-15-02721-s001.zip › 2025-06-21-080738-image5-sub2-As-Displayed.tif]

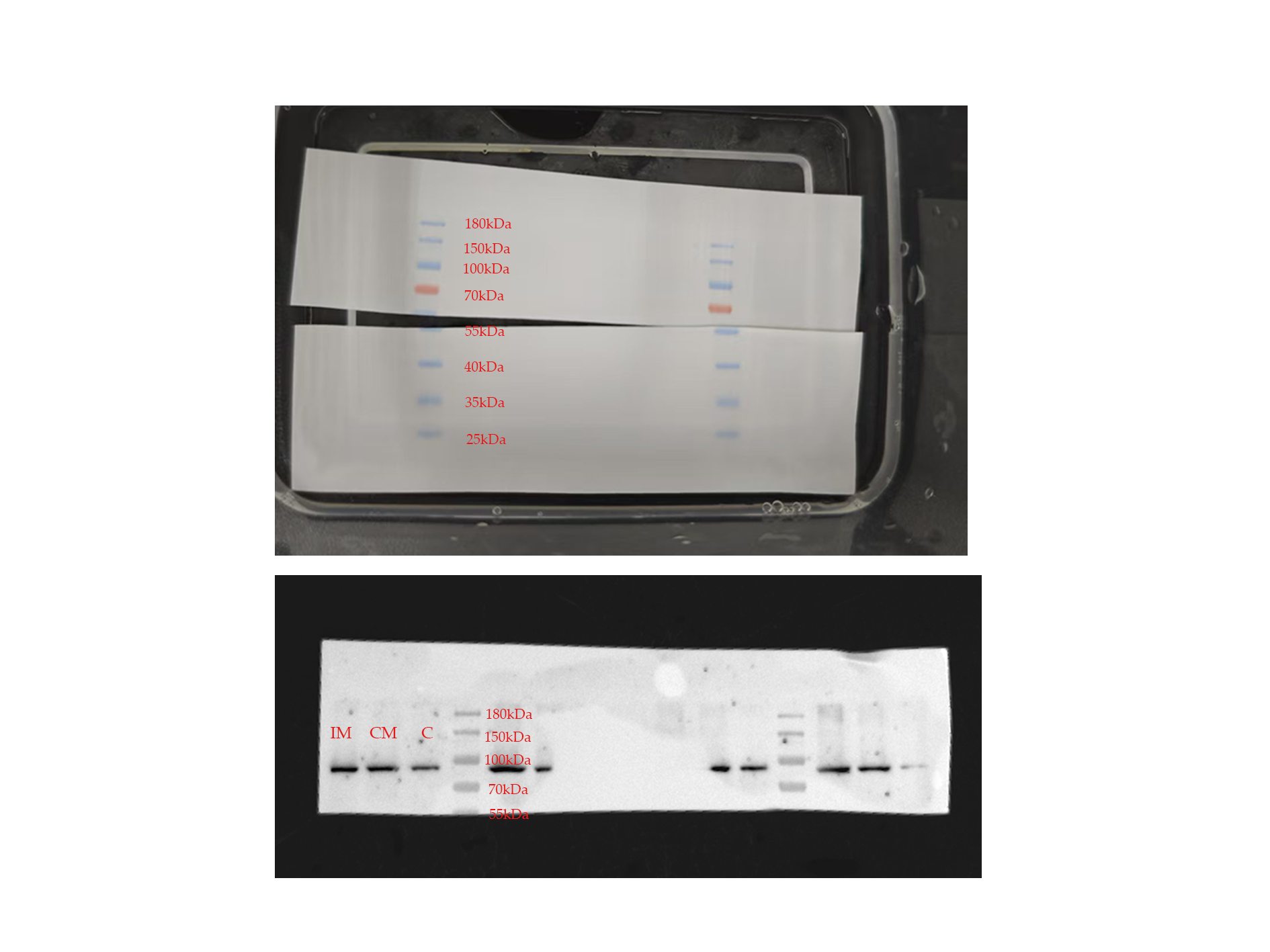

Supplement: Supplementary file 1 [file animals-15-02721-s001.zip › 2025-06-21-080811-image5-83kDa.tif]

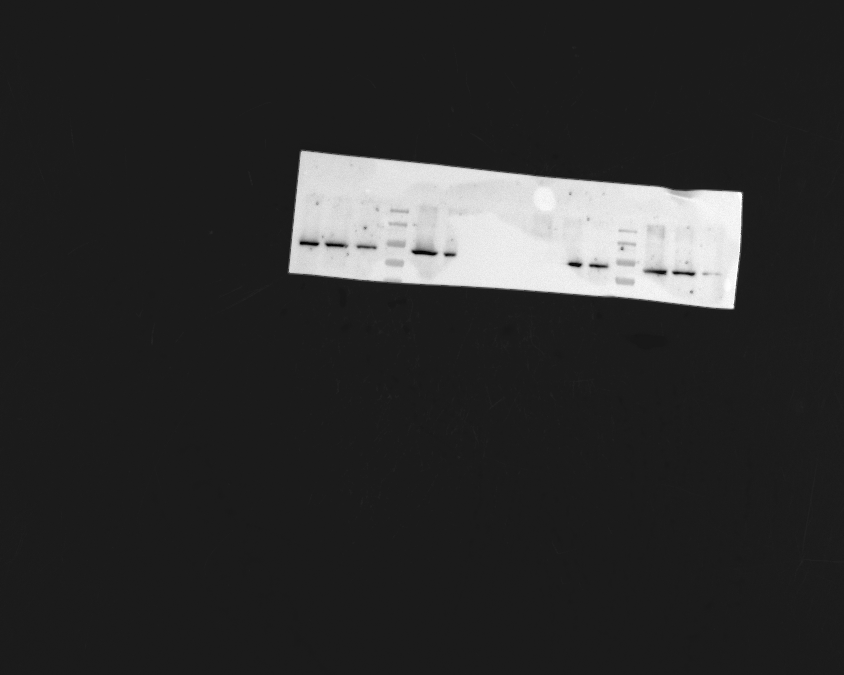

Supplement: Supplementary file 1 [file animals-15-02721-s001.zip › 2025-06-21-080811-image5.tif]

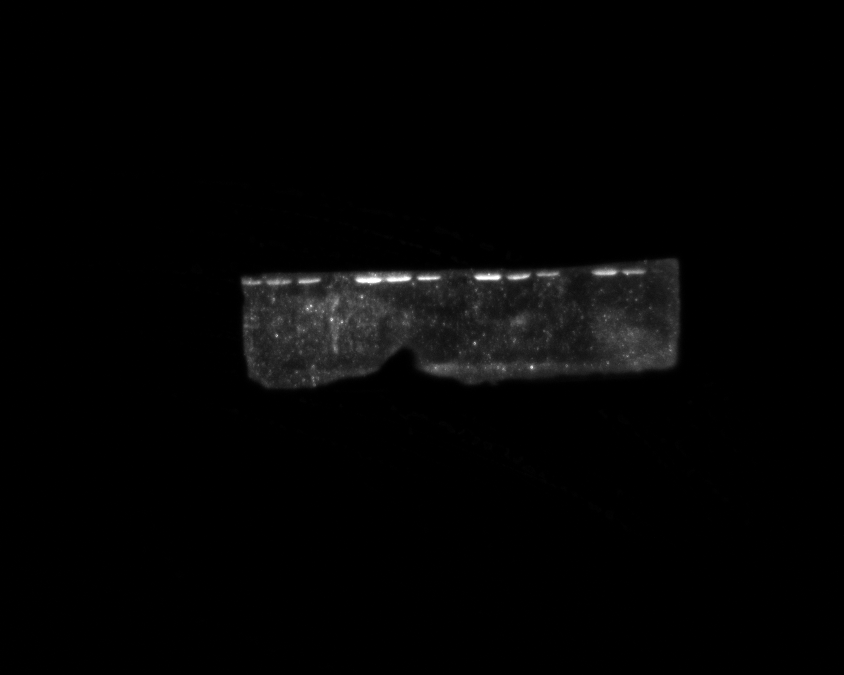

Supplement: Supplementary file 1 [file animals-15-02721-s001.zip › 2025-06-21-081142-image6-sub0-As-Displayed.tif]

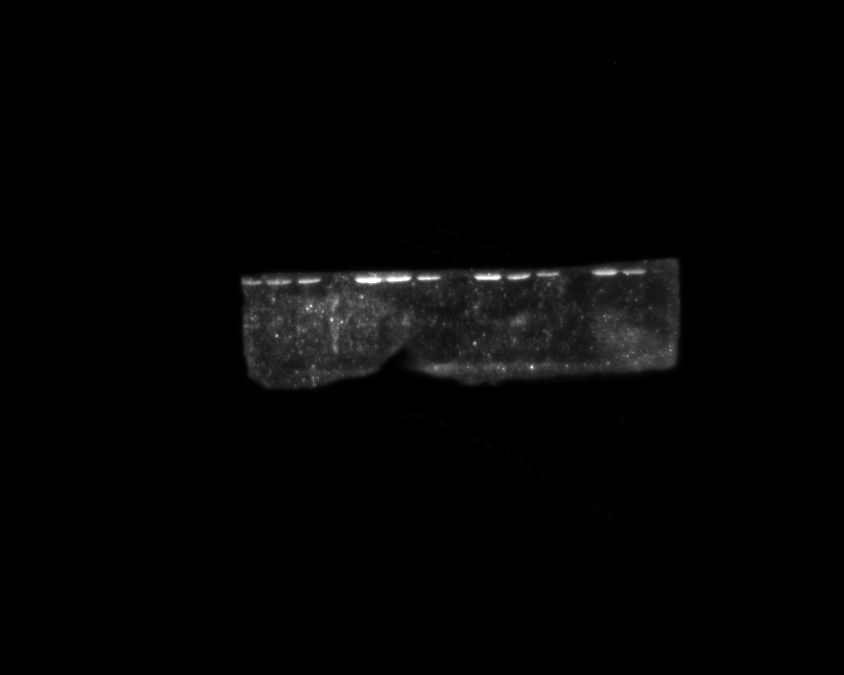

Supplement: Supplementary file 1 [file animals-15-02721-s001.zip › 2025-06-21-081142-image6-sub1-As-Displayed.tif]

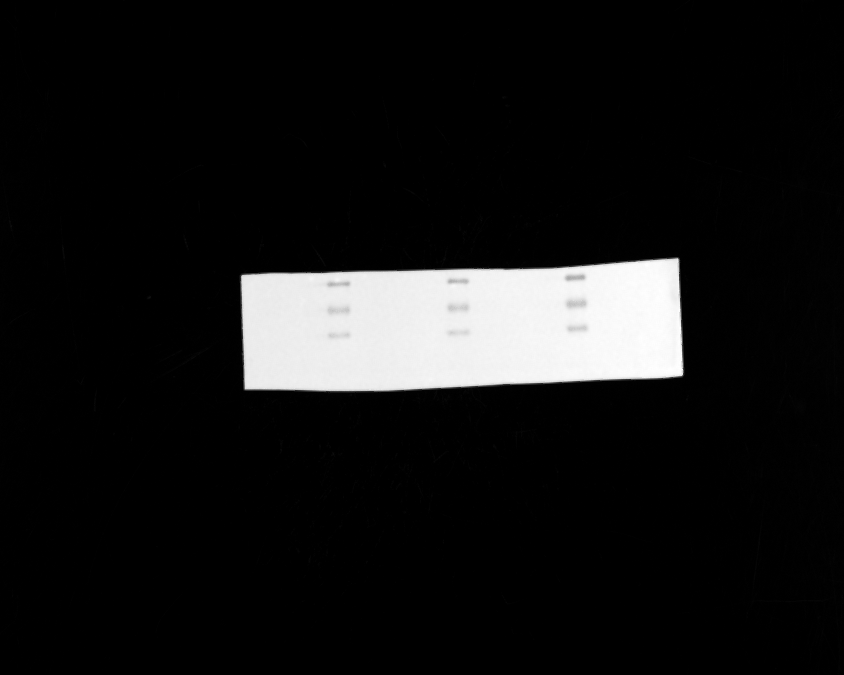

Supplement: Supplementary file 1 [file animals-15-02721-s001.zip › 2025-06-21-081142-image6-sub2-As-Displayed.tif]

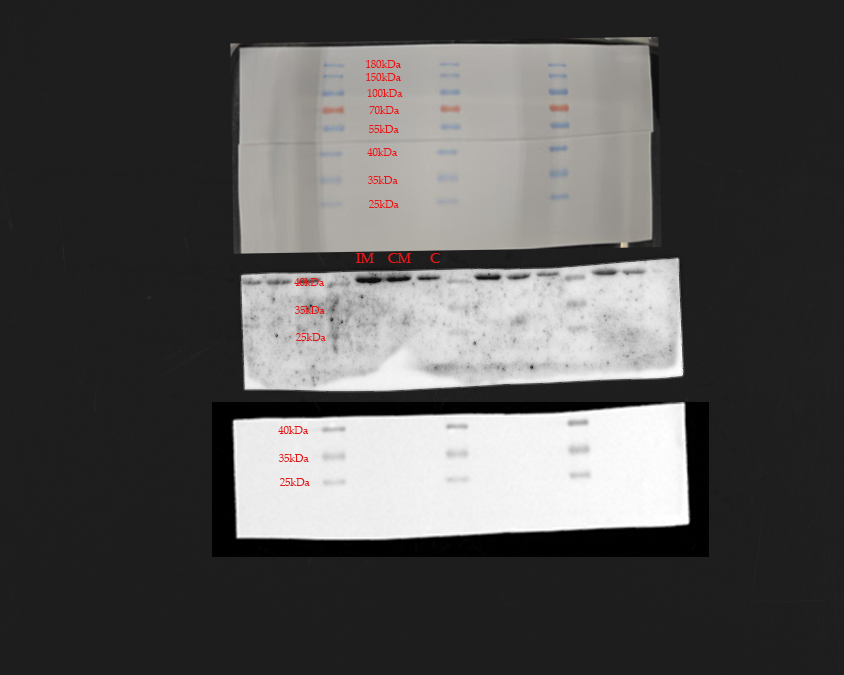

Supplement: Supplementary file 1 [file animals-15-02721-s001.zip › 2025-06-21-081215-image6-45kDa.tif]

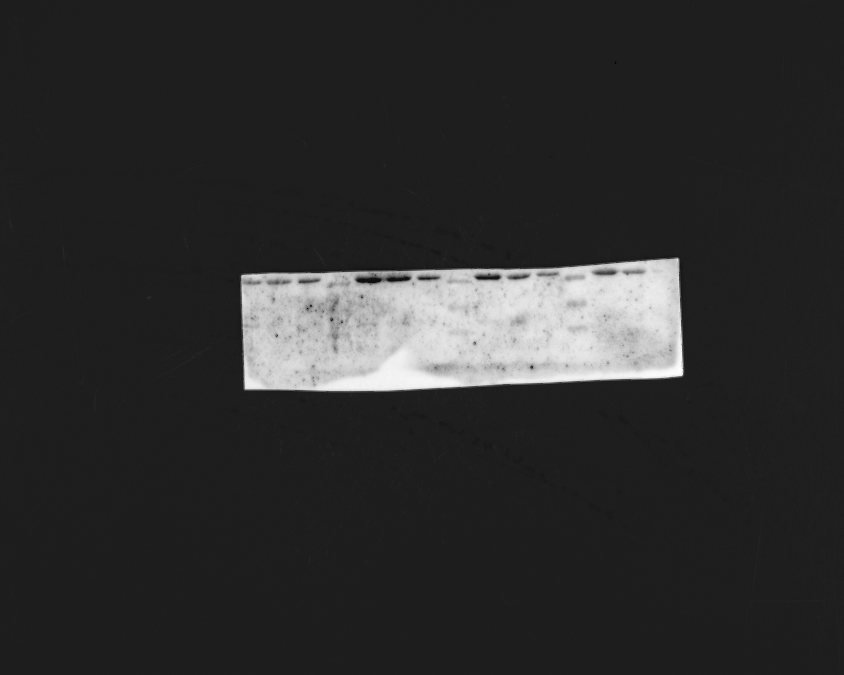

Supplement: Supplementary file 1 [file animals-15-02721-s001.zip › 2025-06-21-081215-image6.tif]

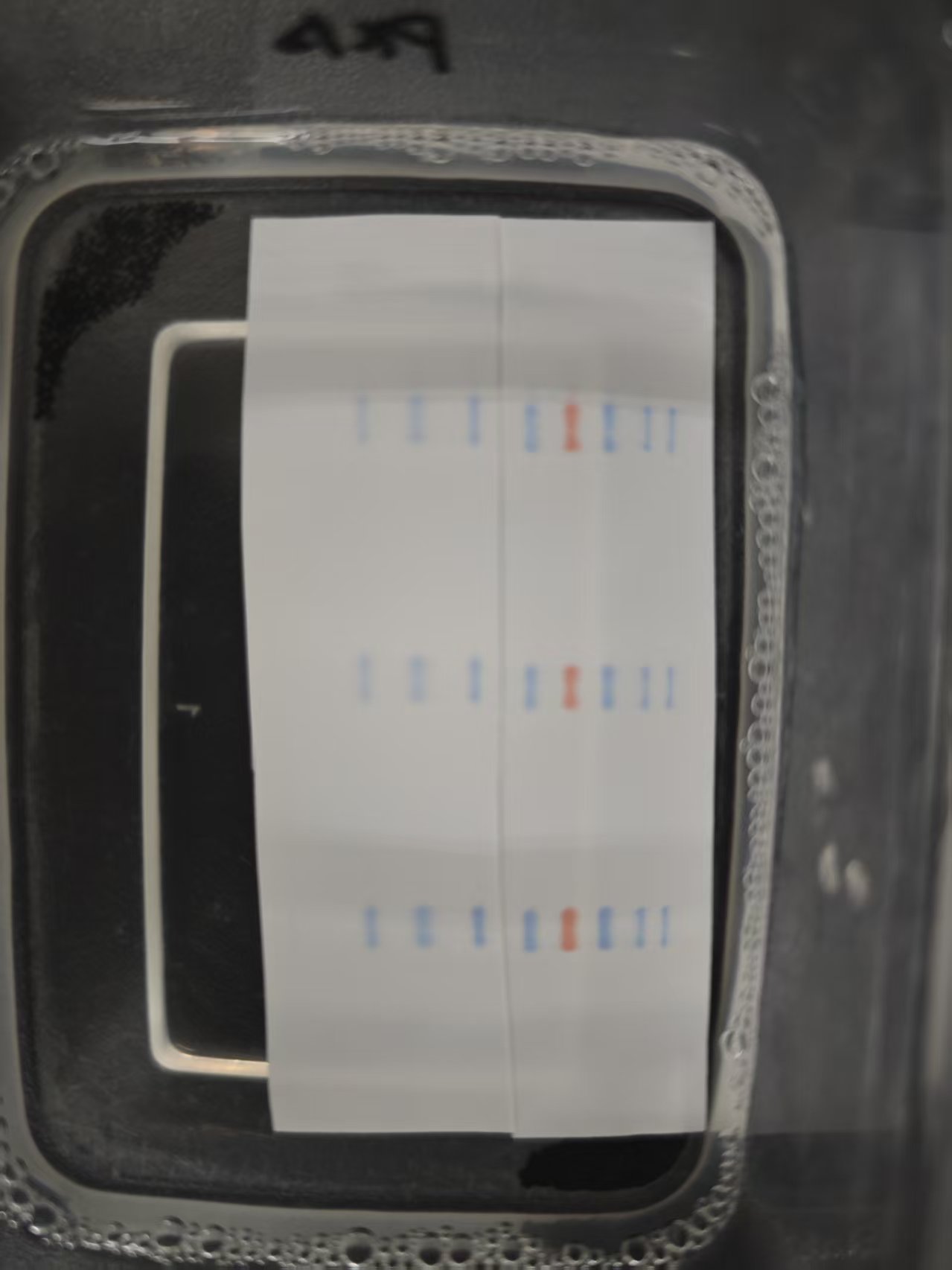

Supplement: Supplementary file 1 [file animals-15-02721-s001.zip › _20250625165121.jpg]

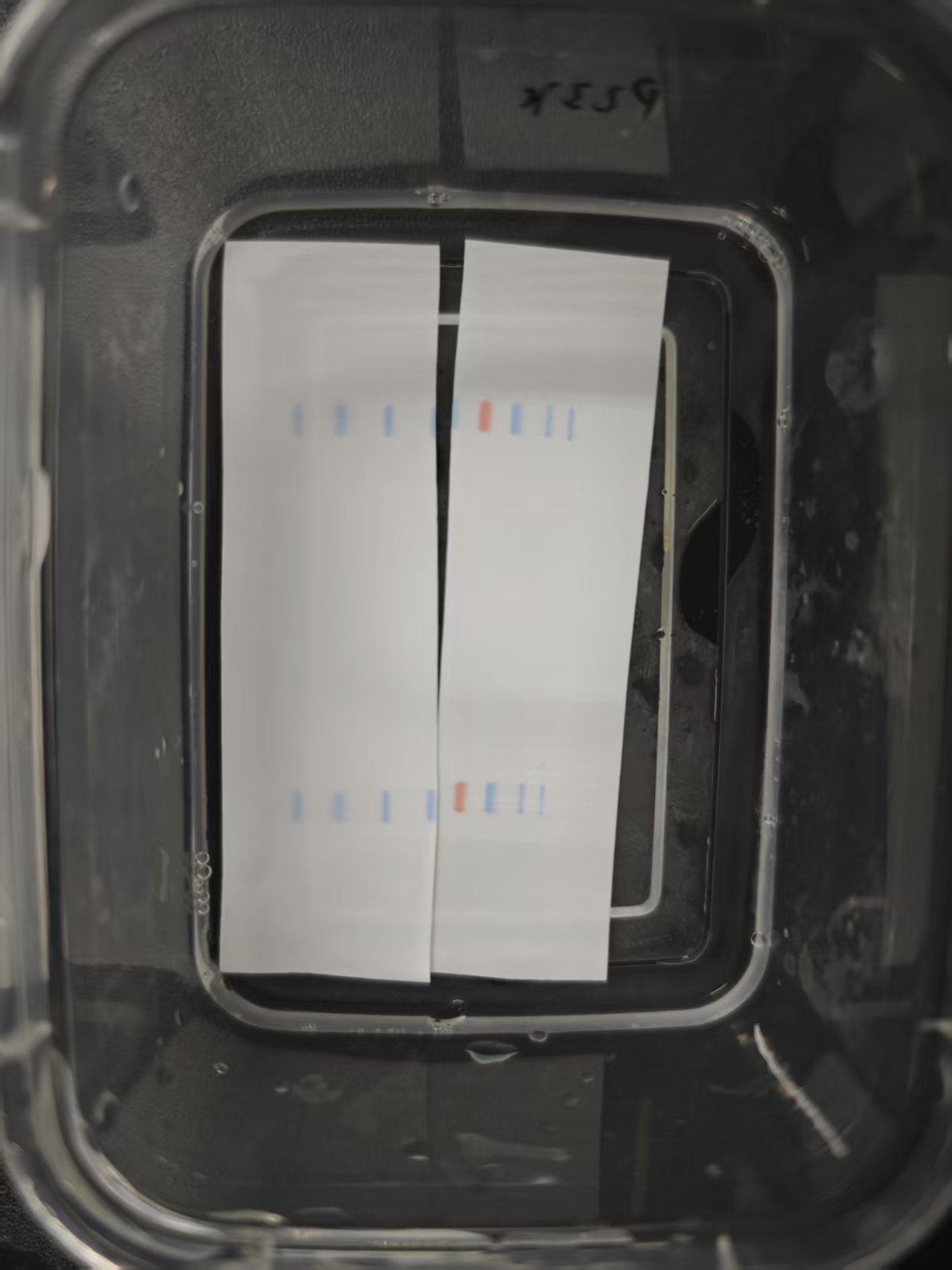

Supplement: Supplementary file 1 [file animals-15-02721-s001.zip › _20250625165305.jpg]
